# Don't eat me/eat me signals as a novel strategy in cancer immunotherapy

**DOI:** 10.1016/j.heliyon.2023.e20507

**Published:** 2023-09-29

**Authors:** Amirreza Khalaji, Fatereh Baharlouei Yancheshmeh, Fatemeh Farham, Arya Khorram, Shiva Sheshbolouki, Maryam Zokaei, Fatemeh Vatankhah, Mehdi Soleymani-Goloujeh

**Affiliations:** a Immunology Research Center, Tabriz University of Medical Sciences, Tabriz, Iran; b Liver and Gastrointestinal Diseases Research Center, Tabriz University of Medical Sciences, Tabriz, Iran; c Cardiac Rehabilitation Research Center, Cardiovascular Research Institute, Isfahan University of Medical Sciences, Isfahan, Iran; d Faculty of Medicine, Tehran University of Medical Sciences, Tehran, Iran; e Department of Laboratory Sciences, School of Allied Medical Sciences, Alborz University of Medical Sciences, Karaj, Iran; f Faculty of Medicine, Hormozgan University of Medical Sciences, Bandar Abbas, Iran; g Department of Food Science and Technology, Faculty of Nutrition Science, Food Science and Technology/National Nutrition and Food Technology Research Institute, Shahid Beheshti University of Medical Sciences, Tehran, Iran; h Department of Veterinary Medicine, Beyza Branch, Islamic Azad University, Beyza, Iran; i Student Research Committee, Tabriz University of Medical Sciences, Tabriz, Iran; j Department of Stem Cells and Developmental Biology, Cell Science Research Center, Royan Institute for Stem Cell Biology and Technology, ACECR, Tehran, Iran

**Keywords:** Cancer, Don't eat me signal, Eat me signal, Immunotherapy, Phagocytosis

## Abstract

Cancer stands as one of the prominent global causes of death, with its incidence burden continuously increasing, leading to a substantial rise in mortality rates. Cancer treatment has seen the development of various strategies, each carrying its drawbacks that can negatively impact the quality of life for cancer patients. The challenge remains significant within the medical field to establish a definitive cancer treatment that minimizes complications and limitations.

In the forthcoming years, exploring new strategies to surmount the failures in cancer treatment appears to be an unavoidable pursuit. Among these strategies, immunology-based ones hold substantial promise in combatting cancer and immune-related disorders. A particular subset of this approach identifies "eat me" and "Don't eat me" signals in cancer cells, contrasting them with their counterparts in non-cancerous cells. This distinction could potentially mark a significant breakthrough in treating diverse cancers. By delving into signal transduction and engineering novel technologies that utilize distinct "eat me" and "Don't eat me" signals, a valuable avenue may emerge for advancing cancer treatment methodologies.

Macrophages, functioning as vital components of the immune system, regulate metabolic equilibrium, manage inflammatory disorders, oversee fibrosis, and aid in the repair of injuries. However, in the context of tumor cells, the overexpression of "Don't eat me" signals like CD47, PD-L1, and beta-2 microglobulin (B2M), an anti-phagocytic subunit of the primary histocompatibility complex class I, enables these cells to evade macrophages and proliferate uncontrollably. Conversely, the presentation of an "eat me" signal, such as Phosphatidylserine (PS), along with alterations in charge and glycosylation patterns on the cellular surface, modifications in intercellular adhesion molecule-1 (ICAM-1) epitopes, and the exposure of Calreticulin and PS on the outer layer of the plasma membrane represent universally observed changes on the surface of apoptotic cells, preventing phagocytosis from causing harm to adjacent non-tumoral cells.

The current review provides insight into how signaling pathways and immune cells either stimulate or obstruct these signals, aiming to address challenges that may arise in future immunotherapy research. A potential solution lies in combination therapies targeting the "eat me" and "Don't eat me" signals in conjunction with other targeted therapeutic approaches. This innovative strategy holds promise as a novel avenue for the future treatment of cancer.

## Highlights

• Modulating "Don't eat me" signals/"Eat me" signals can impede and potentially halt tumor progression.
• Targeting "Don't eat me" signals might enhance the survival rate among cancer patients.
• Enhancing the expression and recognition of "Eat me" signals could aid in eradicating cancerous cells.
• Concurrent therapies addressing "eat me"/"Don't eat me" signals in cancer, alongside other targeted treatments, could emerge as groundbreaking options for future cancer therapy.

## Abbreviations

**ADCP**: Antibody-dependent cellular phagocytosis
**APC**: Antigen-presenting cell
**ATP**: Adenosine triphosphate
**B2M**: Beta-2 Microglobulin or Anti-Phagocytic Subunit of the Major Histocompatibility Complex Class I
**BTK**: Bruton's Tyrosine Kinase
**CAR**: Chimeric Antigen Receptor
**CD**: Cluster of Differentiation
**CRT**: Calreticulin
**EV**: Extracellular Vesicle
**FAK**: Focal Adhesion Kinase
**FcyR**: IgG Receptor
**FcγR**: Fcγ Receptor
**ICAM-1**: Intercellular Adhesion Molecule-1
**ICD**: Immunogenic Cell Death
**Ig**: Immunoglobulin
**ILK**: Integrin-Linked Kinase
**ITAM**: Immunoreceptor Tyrosine-Based Activation Motif
**ITIM**: Immunoreceptor Tyrosine-Based Inhibitory Motif
**ITSM**: Immunoreceptor Tyrosine-Based Switch Motif
**LPC**: Lysophosphatidylcholine
**LRP1**: Lipoprotein Receptor-Related Protein 1
**Mac-1**: Macrophage Antigen 1
**MPS**: Mononuclear Phagocytic System
**NHL**: Non-Hodgkin's Lymphoma
**NK**: Natural Killer Cell
**PC**: Phosphatidylcholine
**PD1**: Programmed Death 1
**PD-L1**: Programmed Cell Death Ligand 1
**PE**: Phosphatidylethanolamine
**PET**: Positron Emission Tomography
**PIP**: Phosphatidylinositides
**PS**: Phosphatidylserine
**S1P**: Sphingosine 1-Phosphate
**SIRPα**: CD47-Signal-Regulatory Protein α
**SLAMF7**: Signaling Lymphocytic Activation Molecule Family Member 7
**SNALP**: Stable Nucleic Acid-Lipid Nanoparticle
**TAM**: Tumor-Associated Macrophage
**TLR**: Toll-Like Receptor
**TNF**: Tumor Necrosis Factor
**TSP**: Thrombospondin

## 1 Introduction

Cancer is a prominent global cause of death, with its incidence burden continuously growing and its mortality rates rising [1,2]. Traditional cancer treatment primarily relied on three main methods: surgery, radiotherapy, and chemotherapy. However, each method comes with inherent issues and drawbacks that can adversely impact the quality of life for cancer patients. Consequently, achieving a definitive cancer treatment with minimal complications and restrictions remains a substantial challenge in medicine [3]. In light of this, many innovative technologies have emerged over the last decade, offering potential solutions for cancer treatment by harnessing the inherent capabilities of natural cells within the body. Novel treatment avenues, including immunotherapy, cellular therapy, gene therapy, and hormone therapy, have been proposed as strategies to overcome these limitations [4]. The field of immunology-based treatments is rapidly expanding, with one subset focusing on identifying "eat me" and "Don't eat me" signals on the surface of cancer cells. This approach involves comparing the presentation of these signals on cancer cells to that of non-cancer cells. Among the critical components of the immune system, macrophages exhibit considerable structural diversity and fulfill diverse roles in metabolic homeostasis, inflammation management, fibrosis processes, and wound healing [5]. Macrophages play a fundamental role in presenting antigens to T cells for cellular immunity, which designates them as "antigen-presenting cells" (APCs) [6]. Recent research has established that inhibitory and stimulatory signals regulate the innate immune system's phagocytosis of malignant cells. Identifying and targeting these signals for novel treatment strategies holds promise [7,8]. Adequate Clearance of pathogenic, dying, or inactive cells relies on phagocytic responses elicited by "eat me" and "Don't eat me" signals displayed on host cell surfaces [9].

Tumoral cells often upregulate "Don't eat me" signals like CD47, programmed cell death ligand 1 (PD-L1), and B2M. These signals allow cancer cells to evade macrophage clearance, leading to uncontrolled proliferation [10]. Conversely, presenting "eat me" signals, such as PS, alterations in surface charge and glycosylation patterns, changes in ICAM-1 epitope, and exposure of Calreticulin and PS on the outer plasma membrane leaflet, are prevalent in apoptotic cells. These changes prevent adjacent non-tumoral cells from being damaged by phagocytes [11].

Therapeutic approaches based on "Don't eat me"/"Eat me" signals modify macrophage phenotypes. By targeting macrophage transcriptional machinery and post-transcriptional regulation, these signals can, directly and indirectly, alter macrophage behavior [12]. These processes affect gene expression and influence cytokine production, leading to shifts in macrophage phenotype [13]. For instance, binding "Don't eat me" signals to macrophages can increase phagocytosis, decrease pro-inflammatory cytokine production, and raise anti-inflammatory cytokine production. These approaches reshape macrophage phenotypes by modifying cell surface molecules and receptors. Such changes enable macrophages to act as primary defenders against infections, potentially enhancing the efficacy of medical treatments.

Additionally, this strategy has effectively reduced inflammation and tissue damage prompted by pathogens. Enhancing macrophages' ability to recognize and counteract pathogens can expedite immune responses, aiding the body in combatting infections. Moreover, this can limit the spread of infection throughout the body and mitigate inflammation and tissue damage associated with infections.

Given the critical significance of "eat me" and "Don't eat me" signals, particularly in cancer pathogenesis, exploring the mechanisms and molecules implicated in these signals can pave the way for improved cancer treatments, focusing on innovative immunotherapeutic approaches. Thus, this review delves into the involved signaling pathways and immune cells that regulate the stimulation or inhibition of these signals. It also seeks to address the challenges that future studies may encounter as they delve deeper into investigating cancer immunotherapy.

## 2 "Eat me" signals and "Don't eat me" signals; who are them, and how do they act?

In the realm of multicellular organisms, the phenomenon of apoptosis, or programmed cell death, plays a pivotal role in various essential processes such as morphogenesis, cellular homeostasis, injury repair, immunological tolerance, and the mitigation of inflammation [14,15]. For instance, during neurogenesis and retinogenesis, tissue remodeling becomes imperative, necessitating the elimination of as much as 50% of surplus neurons through apoptosis [14]. An astonishing fact is that Kupffer cells, the resident macrophages in the liver, are estimated to engulf over 2.4✕10^6^ red blood cells every second [16]. Furthermore, a staggering number of approximately 1✕10^11^ circulating neutrophils are renewed daily [14]. In central immune tolerance, around 95% of maturing T cells undergo elimination within the thymus via negative selection [17]. Throughout our lifespans, which can span up to 80 years, the bone marrow, lymph nodes, and intestines collectively generate up to 2 tons of apoptotic cells [14]. While autologous apoptotic cells and foreign infections can be engulfed through phagocytosis, the resultant immunological effects differ markedly. The phagocytosis of opsonized pathogens, facilitated by complement and Fc receptors, prompts pro-inflammatory responses. Conversely, the ingestion of apoptotic cells triggers anti-inflammatory responses. This specific process is termed efferocytosis, a term derived from the Greek word "Affero," which conveys the idea of conveying to a tomb or burying. The term "engulfment" is used to distinguish between these two processes [18,19].

Specific critical signals emerge to regulate the intricacies of phagocytosis and identify cells suitable for engulfment. These signals, classified into two distinct categories as "eat me" and "Don't eat me" signals, are pivotal in determining whether a cell is recognized as a target for phagocytosis or spared from this fate.

### 2.1 "Eat me" signal

Recent discoveries have unveiled four potential "find me" signals apoptotic cells emit. These signals encompass lipid lysophosphatidylcholine (LPC), sphingosine 1-phosphate (S1P), fractalkine CX3CL1, nucleotides ATP, and UTP. Although diverse, all these molecules are linked to the attraction of macrophages or monocytes toward apoptotic cells [20]. Despite the assistance these "find me" signals provide in guiding phagocytes to the vicinity of the dying cell, an essential challenge remains: the phagocyte must accurately discern the dying cell from the multitude of viable cells. To achieve this, specific markers known as "eat me," signals must be presented on the surface of apoptotic cells. This recognition process is analogous to identifying a particular house amidst a row of residences, much like spotting the fire amidst a complex scenario. The "find me" signals operate akin to a fire alarm that directs a fire rescue team to the precise location of a house on fire (in this context, the "eat me" signals). Notably, numerous "eat me" signals are visibly displayed on the surface of apoptotic cells. These include PS exposure, modifications to the cell surface's charge and glycosylation patterns, alterations to the epitopes of ICAM-1, and the presence of Calreticulin—a protein in the endoplasmic reticulum [21,22]. Among the mentioned agents, PS exposure primarily stands out as the key trigger for the "eat me" signal.

Compared to PS, predominantly found on the outer leaflet of the plasma membrane, phosphatidylcholine (PC) and sphingomyelin exhibit greater prevalence. Conversely, phosphatidylethanolamine (PE) and phosphatidylinositides (PIPs) are typically confined to the cytoplasmic leaflet. This asymmetric distribution of phospholipids across the inner and outer leaflets of the bilayer significantly shapes the biophysical properties of the membrane, playing a pivotal role in cell signaling and regulation [23]. While the primary exception lies in the externalization of PS as an "eat me" signal during apoptotic cell elimination via efferocytosis, instances of alterations in phospholipid asymmetry are infrequent [24].

### 2.2 "Don't eat me" signal

While most living cells do not exhibit signals indicating "eat me," certain physiological situations, such as lymphocyte activation, skeletal muscle development, and sperm-egg fusion, prompt some living cells to temporarily present markers resembling those of a dying cell [9]. These "don't eat me" signals are consequently expressed as an additional regulatory mechanism to prevent the inappropriate removal of viable cells. Most well-defined anti-phagocytic receptors are single-pass, type I transmembrane proteins belonging to the immunoglobulin (Ig) superfamily. They possess one or more immunoreceptor tyrosine-based inhibitory motifs (ITIM) within their cytoplasmic tails [25]. The conventional ITIM consensus sequence is (I/V/L) xYxx(L/V), with x representing any amino acid. Numerous anti-phagocytic receptors also incorporate immunoreceptor tyrosine-based switch motifs (ITSM), supplementary protein-binding domains, and non-canonical "ITIM-like" motifs featuring a more diverse sequence (I/V/L/SxYxxL/V/I) [25,26]. Activation of anti-phagocytic receptors triggers tyrosine phosphorylation of the cytoplasmic ITIMs, leading to the activation of cytosolic SH2 domain-containing phosphatases. These include the SHP tyrosine phosphatases and the SHIP1/2 inositol phosphatases [25,27]. Although identifying their downstream substrates has proven challenging, these phosphatases function as downstream effectors, facilitating the inhibitory role of anti-phagocytic receptors. For instance, the inhibitory receptor FcγRIIb (Fcγ receptors Iib) plays a crucial regulatory role in antibody-dependent cellular phagocytosis (ADCP) of opsonized targets. The achievement of this process is facilitated through the activation of SHIP1/2 [28,29]. Like most other anti-phagocytic receptors, the inhibitory function of these receptors relies on the SHP-1 and/or SHP-2 tyrosine phosphatases [29]. Structurally, SHP-1 and SHP-2 comprise a catalytic phosphatase domain, a C-terminal tail, and two N-terminal SH2 domains [30]. While the precise molecular intricacies remain partly unclear, it is believed that the phosphorylation of two consecutive ITIMs is necessary for the binding and activation of SHP downstream of anti-phagocytic receptors [29,30].

## 3 Phagocytosis as a potential avenue for cancer immunotherapy

### 3.1 Utilizing "eat me" signals in cancer immunotherapy

Leveraging the "eat me" signal within cancer immunotherapy has become a captivating subject for researchers due to its considerable potential and wide-ranging implications. Various strategies are being explored for integrating the "eat me" signal into immunotherapy to enhance the effectiveness and efficiency of therapeutic agents within the patient's body. A prominent challenge in cancer treatment is that a substantial proportion, up to 99%, of nanoparticles are eliminated within the liver, irrespective of their size and shape [31,32]. This phenomenon can be attributed to tissue-resident macrophages identifying, attacking, capturing, and eliminating foreign entities [33,34]. One viable approach to harness the "eat me" signal involves employing extracellular vesicles (EVs) as carriers of this signal. The Zakia Belhadis group has enhanced macrophage targeting by functionalizing EVs with cationized mannan, thereby reducing mononuclear phagocytic system (MPS) sequestration. This optimization subsequently augments the accumulation of these vesicles in tumors, bolstering their therapeutic efficacy against lung cancer. Encouragingly, these modifications have demonstrated no adverse impacts on the liver or spleen, the primary organs responsible for foreign material clearance [35].

An alternative strategy to capitalize on the "eat me" signal entails augmenting phagocytosis by rendering target cells more discernible to macrophages and other immune system components. Cancer cells often deploy both "eat me" and "Don't eat me" signals to evade phagocytic cell destruction. A growing body of evidence indicates that accentuating antigen presentation and leveraging the impact of macrophages and dendritic cells on tumors could substantially complement conventional treatments like chemotherapy and radiation [36]. Within the realm of tumor immunogenic cell death (ICD), which constitutes a form of apoptosis [37] ], a pivotal role is played by calreticulin translocation to the cell surface, acting as an "eat me" signal [38]. During ICD, Calreticulin interacts with low-density lipoprotein receptor-related protein 1 (CD91) in phagocytes [39]. While drugs can elevate surface calreticulin levels to prompt ICD [40], it is noteworthy that CD47 expression counterbalances and mitigates Calreticulin's effect [41]. In a study by Hend Mohamed Abdel-Bar and colleagues, the researchers explored the development of a stable nucleic acid-lipid nanoparticle (SNALP) system. This system co-delivered ICD-inducing drugs (such as doxorubicin) and siRNA to inhibit CD47. The findings underscore the potential impact of simultaneously employing these two formulations in treating specific types of immunogenic solid tumors. The approach enhanced CD8^+^ cell infiltration and activated systemic immune system [38].

A related endeavor by Suchismita Mohanty and their team sought to amplify the efficacy of immunotherapy by harnessing the "eat me" signal [42]. Tumor-associated macrophages (TAMs) can be harnessed in immunotherapeutic interventions [43], offering the capability to engage tumor cells directly [44]. Given Doxorubicin's capacity to trigger immunogenic cell death [45], characterized by heightened calreticulin expression on target cell surfaces [41], Suchismita Mohanty investigated the impact of combining doxorubicin with CD47 monoclonal antibodies (mAb). CD47 mAb blocks CD47 receptors on cell membranes [46,47]. The results of this study emphasized the potential positive interaction between blocking the anti-phagocytic signal and enhancing the pro-phagocytic signal in the context of osteosarcoma immunotherapy [42].

Beyond therapeutic interventions, nanomaterials like iron oxide hold promise for medical imaging, including techniques like MRI and Positron Emission Tomography (PET) [48]. An additional avenue for leveraging the "eat me" signal within immunotherapy lies in analyzing treatment processes and imaging sites exhibiting drug activity and function [49]. Phosphatidylserine (PS), a constituent of cell membranes, functions as an "eat me" signal during the apoptosis phase [50,51] (Fig. 2). Bagalkot's work highlights the potential of synthetic PS designed for targeting tumor cells, not only in facilitating drug delivery but also in visualizing phagocytosis [49].

### 3.2 Unveiling the "Don't eat me" signal in cancer immunotherapy

The liver is the habitat with the largest concentration of tissue-resident macrophages [52]. These macrophages are responsible for capturing and eliminating foreign substances [34]. Apoptosis, a programmed cell death mechanism, exhibits multifaceted attributes contributing to its intricate and captivating nature [53]. Phagocytes detect and engulf dying cells before intracellular components are released, preventing the exposure of immunogenic materials and subsequent inflammatory responses [54]. However, this process is inherently physiological; its disruption or dysregulation can link to pathological conditions, possibly catalyzing certain diseases [19]. Eliminating aberrant or diseased cells is indispensable for proper development, tissue integrity, and defense against pathogenic and immunogenic harm [55]. Both professional and non-professional phagocytes participate in apoptosis [56]. Among the professional phagocytes are macrophages and immature dendritic cells, known for their relatively swift responses. In contrast, non-professional phagocytes consist of tissue-resident cells near dying cells, showcasing a sluggish ability to ingest deceased cells [57,58]. Innate immune cells are deemed professional phagocytes due to their capacity to recognize and migrate toward target cells, ingesting multiple targets [59]. For the protection of healthy cells, a controlled phagocytic process is essential. This process unfolds across four phases: "smell," "taste," "ingestion," and "digestion/response." In the "taste" phase, an "integrated" recognition of both "eat me" and "Don't eat me" signals is necessary, as both are expressed on the target cell. "Eat me" signals encompass antibody and complement opsonins, exposed PS, Calreticulin, oxidized low-density lipoprotein, cell-bound thrombospondin (TSP), and other surface protein modifications [55]. Ordinarily, living normal cells abstain from expressing "eat me" signals, though exceptions exist in physiological contexts, where specific living cells transiently display this signal, as seen during lymphocyte activation, skeletal muscle formation, and sperm-egg fusion [60,61]. Consequently, the presence of "Don't eat me" signals on healthy cell surfaces protects against unwarranted Clearance. In the realm of cancer cells, the "Don't eat me" signal is exploited to evade detection by the innate immune system, effectively eluding elimination by initial responder cells such as macrophages.

**Fig. 1:**
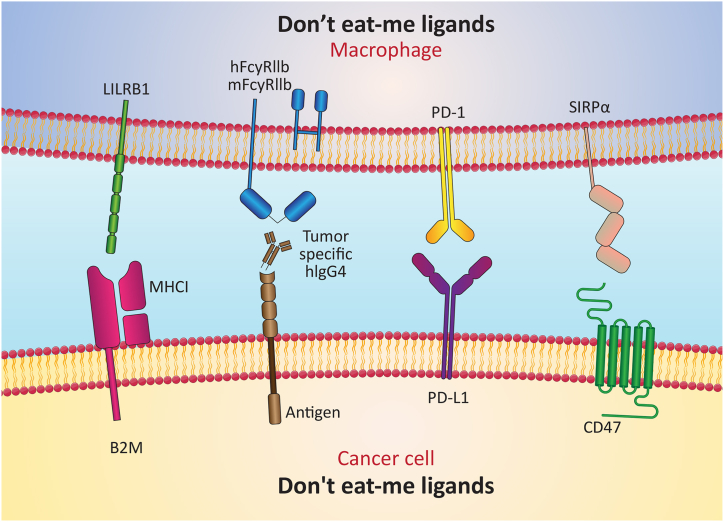
Schematic illustration depicting significant ligands for the "Don't eat me" signal compared to corresponding receptors.

Discovered in 1990 as a protein interacting with integrins, CD47 stands as a glycoprotein [62]. Its structure comprises an extracellular N-terminal single immunoglobulin V-like domain featuring multiple glycosylation sites, five membrane-spanning regions, and a C-terminal intracellular domain [63]. CD47's interaction partner is CD47-Signal-regulatory protein α (SIRPα) [64]. SIRPα showcases three extracellular immunoglobulin-like domains, succeeded by a lone transmembrane segment, ultimately leading to an intracellular signaling domain equipped with ITIM [65]. THROUGH ITS SIGNALING, the CD47/SIRPα axis contributes to several physiological homeostatic processes [64]. While normal tissues exhibit modest CD47 expression, tumors commonly exhibit overexpression [66]. The elevation of CD47 protein expression by cancer cells serves as a mechanism to sidestep immune-driven eradication [67]. Notably, CD47 emerges as a potent "Don't eat me" signal, and its excessive presence typifies solid and hematological tumors, encompassing acute leukemia, non-Hodgkin's lymphoma (NHL), colorectal cancers, and ovarian cancers. In numerous malignancies, this upregulation aligns with an overall unfavorable prognosis [9,63]. The inhibition of CD47 signaling can incite macrophage phagocytic activity, culminating in impaired tumor growth, the suppression of metastatic spread, and even tumor regression. Notably, the inhibition of CD47 signaling is an efficacious strategy, underscored by the recent emergence of several CD47-targeting agents entering clinical trials [63] Another noteworthy "Don't eat me" signal stems from CD24, which interacts with the inhibitory receptor known as sialic acid-binding Ig-like lectin 10 (Siglec-10) on tumor-associated macrophages (TAMs). A host of tumors exhibit CD24 overexpression, particularly ovarian and breast cancer. Notably, administering anti-CD24 antibodies significantly reduced tumor growth within murine models [10]. Therefore, directing attention toward targeting the "Don't eat me" signal emerges as a novel avenue within the landscape of cancer immunotherapies [68] (Fig. 1) Table 1 represents the current status of drug development related to eat me/Don't eat me signals and the progress of associated clinical trials in the future. "Don't eat me" signals are blocked by monospecific antibodies, small molecules, and peptides to promote macrophage phagocytosis. The generally favorable toxicity profile of anti-CD47 therapy, thus, offers promise for ongoing clinical trials of phagocytosis checkpoint blockade in multiple hematological and solid tumors, either as a single agent or in combination with other monoclonal antibodies or T cell immune checkpoint inhibitors. The interaction between CD24 and Siglec-10 mediates immune escape. By inhibiting the connection between CD24 and Siglec-10 using monoclonal antibodies (mAbs), the tumor is effectively phagocytosed. Novel blockers and different biomolecules are developing for targeted therapy of "Don't eat me"/"eat me" signals in cancer therapy that insist their prominent role in ccancer therapy clinical trials. Novel blockers and different biomolecules are developed for targeted therapy of "Don't eat me"/"eat me" signals in cancer therapy that emphasize their prominent role in cancer therapy clinical trials.

**Fig. 2:**
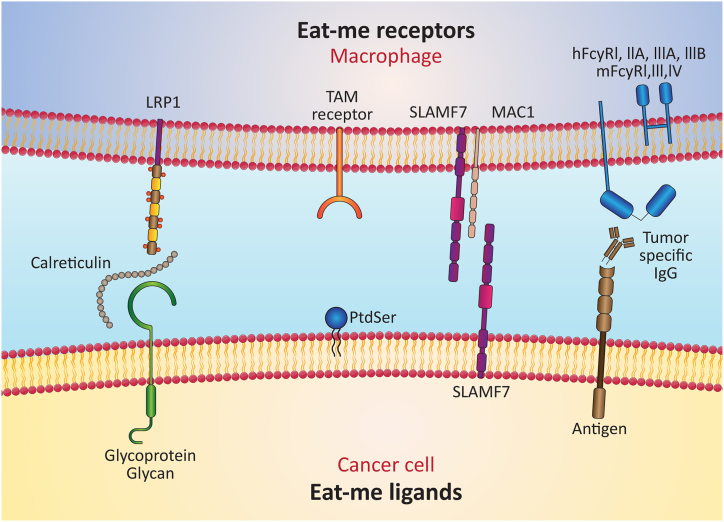
Schematic representation showcasing key ligands involved in the "Eat me" signal and their respective receptors.

**Table 1:** The current status of drug development related to eat me/Don't eat me signals and the progress of associated clinical trials in the future. "Don't eat me" signals are blocked by monospecific antibodies, small molecules, and peptides to promote macrophage phagocytosis.

Clinical Trial(s) ID	Trial Phase	Target	Drug	Pathologies
NCT04313881 NCT04778397 NCT05079230 NCT04788043 NCT04827576 NCT04958785 NCT04892446 NCT04854499 NCT04778410 NCT04541017 NCT04435691 NCT02953782 NCT02953509 NCT05169944 NCT04751383	III III III II II II II II II I + II I + II I + II I + II I I	CD47	Magrolimab	AML
NCT05002127 NCT04675294 NCT04675333 NCT05167409 NCT04755244 NCT04417517 NCT05025800 NCT05027139 NCT03013218	II + III II II II I + II I + II I + II I + II I	CD47	Evorpacept (ALX148)	B cell non- Hodgkin lymphoma
NCT04886271 NCT04445701 NCT03834948 NCT04445701	I + II I + II I + II I + II	CD47	AO-176	Malignancies/multiple myeloma Advanced solid tumors, relapsed/refractory multiple myeloma
NCT04900350 NCT04980885 NCT04349969 NCT04728334	I + II I + II I I	CD47	AK117	AML/myelodysplastic syndrome, malignant neoplasm
NCT04097769	I	CD47	HX009	Advanced solid tumors
NCT04266301 NCT04823624 NCT04150029 NCT04878432 NCT03946670 NCT04812548 NCT04623216 NCT02608268	III II II II II II I + II I + II	Tim4/3	Sabatolimab	Cancers: advanced malignancies/lower risk myelodysplastic syndrome/AML/myelodysplastic syndromes
NCT03680508 NCT0281763 NCT03307785	II I I	Tim4/3	TSR-022	Advanced solid tumors/liver cancer/melanoma
NCT03099109	I	Tim4/3	LY3321367	Advanced/refractory solid tumors
NCT03824080 NCT03184571 NCT03654833 NCT03649321 NCT03965494 NCT02488408	II II II I + II I I	Axl	Bemcentinib	Recurrent glioblastoma, advanced solid tumors, metastatic breast cancer, AML, non- small cell lung cancer, malignant mesothelioma, Non-small cell lung cancer
NCT01357395	II	Axl	Amuvatinib	Small cell lung cancer
NCT01639508	II	Axl	Cabozantini	Non-small cell lung cancer
NCT02729298	I	Axl	TP-0903	Advanced solid tumors
NCT05888701	I	CD24	anti-CD24 mAb	Mantle-cell Lymphoma (MCL), CLL
NCT04552704	I + II	CD24	CD24Fc	advanced solid tumors
NCT04060407	I + II	CD24	CD24Fc with ipilimumab and nivolumab	Metastatic Melanoma
NCT04552704 NCT04095858 NCT04976699 NCT02663622	I + II III II	CD24	Efprezimod Alfa (CD24Fc, MK-7110)	Solid tumors, Graft-versus-host disease

## 4 Regulation of phagocytic signaling pathways in macrophages

In combating cancer cells, innate immunity emerges as a pivotal player, with macrophages taking center stage in this intricate process [69]. Macrophages collaborate with other constituents of the innate immune system to discern receptors and surface molecules exhibited by infectious or malignant cells, subsequently initiating phagocytosis [70]. These events are orchestrated by diverse signals, categorized into distinct groups.

**Fig. 3:**
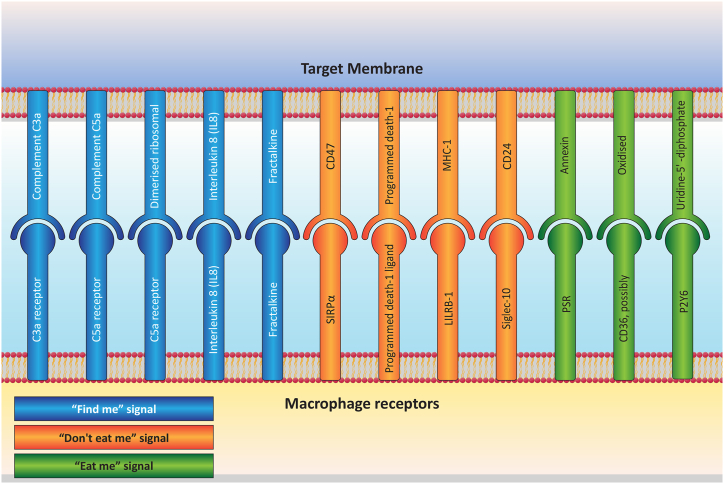
Comprehensive schematic presentation delineating the signaling of "Find me," "Eat me," and "Don't eat me" signals, elucidating macrophage reactivity towards target cells.

The foremost classification, designated as "find me" signals [71], encompasses attraction cues that facilitate the recruitment of macrophages toward cancer cells. Upon reaching the vicinity of cancer cells, these cells emit signals to beckon phagocytosis, termed "eat me" signals [72]. Conversely, specific cells thwart phagocytosis by expressing "Don't eat me" signals [73]. A noteworthy facet of cancer cells lies in their propensity to amplify the expression of both "eat me" and "Don't eat me" signals within their signal interactions. The ensuing interactions can be outlined as follows (See Fig. 3).

### 4.1 CD47-signal-regulatory protein α

The SIRPα family consists of three members: α, β, and γ. Among these subtypes, the interaction between the alpha type and CD inhibits macrophage selection of cancer cells for phagocytosis, allowing them to evade this process. This interaction serves as a "Don't eat me" signal and hampers phagocytosis [74]. Cancer cells display higher levels of CD47 on their surface than non-malignant cells. Numerous studies have demonstrated CD47's overexpression in various tumor types, including myeloma, leiomyosarcoma, acute lymphocytic leukemia, non-lymphoma, Hodgkin's breast cancer, osteosarcoma, and head and neck squamous cell carcinoma [75]. By exploiting the "Don't eat me" signal, cancer cells utilize CD47's overexpression to evade immune cell surveillance and clearance, making CD47 a potential target for novel anti-tumor therapies [76].

Nevertheless, anti-CD47 medications exert their anti-tumor effects through distinct pathways [77]. For instance, they disrupt the CD47-SIRP inhibitory signaling, enhancing macrophage-mediated phagocytosis of tumor cells. In scenarios where targeted cell clearance is increased while avoiding the Clearance of vital cell types, inhibiting the CD47-SIRP axis emerges as an appealing treatment option. Currently, inhibitors of the CD47-SIRP signaling pathway fall into three categories at various stages of development: molecules that obstruct pathway activity by targeting CD47 molecules on target cells, molecules that obstruct pathway activity by targeting SIRP molecules on immune effector cells, and inhibitors of the QPCTL enzyme essential for CD47 maturation [78].

CD47 features an extended N-terminal extracellular domain and 5 transmembrane helices, interacting with SIRPα [79,80] to serve as a "Don't eat me" signal to macrophages. Interfering with this interaction allows the phagocytosis of live cancer cells. Multiple strategies are employed to achieve this, including antibodies targeting CD47 or SIRPα binding sites [81,82], recombinant proteins competing with endogenous proteins for CD47 or SIRPα binding [83,84], and targeting the pathways regulating CD47 transcription to suppress its surface expression on cancer cells [85]. Notably, CD47 is the primary "Don't eat me" ligand, while its receptor on macrophages, SIRPα, inhibits myosin II polymerization—a pivotal step in initiating cell engulfment.

### 4.2 PD-1/PD-L1

Another "do not consume me" signal is presented in tumor cells to prevent their engulfment by macrophages, especially tumor-associated macrophages (TAMs) [86]. Besides macrophages, PD-L1 expression is also observed in various other cell types. Numerous studies have extensively investigated the correlation between PD-L1 expression in macrophages and the prognosis of cancer patients. Considering the generic ligand-receptor interaction, one might argue that the role of the PD-L1/PD-1 binding remains consistent in both macrophages and T cells. However, Singhal et al. contradicted this notion by demonstrating that PD-L1 expressed on macrophages did not suppress the T cell response; instead, it shielded macrophages from T cell-mediated destruction [87]. This finding contrasted with the effects of PD-L1 expression in tumor cells.

Given that PD-L1 and PD-1 form a pair of ligand receptors, the impact of PD-L1 acting as a receptor on host macrophages upon interaction with its ligand PD-1 may have been overlooked. This phenomenon is due to the conventional perception of PD-1 as the primary receptor. A study revealed that after treatment with PD-L1 antibodies, macrophages expressing PD-L1 displayed increased size and heightened activity. Moreover, their proliferation and survival capacities were enhanced. Similarly, treatment with soluble CD80 (sCD80) and soluble PD-1 (sPD-1) resulted in macrophages with larger size, altered morphology, and elevated production of CD86, major histocompatibility complex (MHC) II, and TNF-α. However, the impact of sCD80 treatment surpassed that of sPD-1 treatment.

Additionally, the PD-L1 signal suppressed the mTOR pathway in macrophages, inducing changes in their transcriptome [88]. Based on these observations, PD-L1 appears to play a regulatory role in macrophage proliferation and activation. Another study indicated that inhibiting PD-L1 production in macrophages reduced the expression of M2 indicators IL-10 and arginase-1, coupled with increased expression of M1 markers IL-12 and TNF-α [89].

### 4.3 MHC I-LILRB1

MHC I is a molecule in numerous body cells, and tumor cells also display this molecule as a strategy to evade phagocytosis. The receptor for this molecule on macrophage surfaces is LILRB1 [90]. A gene on chromosome 19q13.42 is accountable for encoding proteins belonging to the leucocyte immunoglobulin-like receptor (LILR) family. The LILR family members possess extracellular immunoglobulin-like domains. Within the LILR family, the activating receptors belong to the subfamily class A LILR receptors, known as LILRA1-5, due to their inclusion of immunoreceptor tyrosine-based activating motifs (ITAMs).

Conversely, the subfamily B LIR receptors, called LILRB1-7, contain multiple cytoplasmic immunoreceptor tyrosine-based inhibitory motifs (ITIMs) [91]. The LILRB subfamily is expressed in cells of myeloid origin, dendritic cells (DCs), B cells, subsets of natural killer (NK) cells, and T cells, giving it a more widespread distribution than other LILR family members. The LILRB receptors comprise three components: an extracellular Ig-like domain, a transmembrane domain, and a cytoplasmic domain housing the ITIM sequences. MHC class I molecules binding to LILRB1 require α3 domains and the β2m subunit [92]. Treatment with type I interferon can enhance the affinity of the LILRB1 protein for HLA-I dimers. LILRB1 exhibits a strong inclination for binding to HLA-I dimers (IFN). The extent of HLA-I expression does not correlate with the level of binding [93]. On T cells, the expression of LILRB1 can compete with CD8 for binding to MHC class I molecules, necessitating the presence of β2m for binding HLA to the LILRB1 receptor. Due to LILRB1's higher affinity for MHC class I and a slower dissociation rate compared to CD8, the combination of MHC class I and LILRB1 is capable of transmitting inhibitory signals to T cells, unlike the MHC class I and CD8 combination. The presence of ITIMs in the cytoplasmic tail of LILRB1 is in harmony with these findings, suggesting that the interaction between MHC class I and LILRB1 is a constituent of an immunological pathway that suppresses immune responses [94].

### 4.4 CD24-Siglec-10

Another noteworthy component of the "don't engulf me" signals is the CD24 molecule, which experiences an elevated expression in tumor cells found in organs such as the ovaries and breasts, prompting tumor-associated macrophages to express its corresponding ligand, Siglec-10 [10]. By employing gene knockout techniques targeting CD24 and Siglec-10, along with monoclonal antibodies designed to obstruct these molecules, researchers have observed a remarkable enhancement in macrophages' capability to engulf tumors. As a result, a deceleration in the growth of macrophage-dependent tumors has been observed in vivo [95]. This significant finding surfaced from the outcomes of experiments that employed gene knockout methodologies to neutralize CD24 and Siglec-10 and monoclonal antibody interventions against these molecules.

Furthermore, the suppression of CD24 generated a response from all macrophages expressing Siglec-10, with the intensity of these reactions correlated to the presence of Siglec-10. The decline in CD24 inhibition was shown to stem from the absence of Siglec-10. These findings indicate a connected sequence of events involving CD24 and Siglec-10 that ultimately leads to the specific blockade of CD24. Unlike Siglec-3 and Siglec-5, CD24 has been demonstrated to exhibit a distinct binding affinity for Siglec-10 [96]. The initiation of the inhibitory signal cascade is brought about by the interaction between CD24 and Siglec-10 [97]. Upon binding, Siglec-10 activates proteins containing the SH2 domain, most notably SHP-1, SHP-2, or suppressor of cytokine signaling 3, once the SRC family tyrosine kinases phosphorylate the intracellular tyrosine-based signal transduction cluster (SOCS3). An essential member of the tyrosine phosphatase family, SHP-1 selectively attaches to phosphorylated tyrosine residues within the intracellular ITIM domain and subsequently catalyzes their dephosphorylation. Furthermore, it modulates intracellular signal transduction [98], encompassing growth factors, cytokines, hormones, extracellular matrix, and cell adhesion molecules.

Consequently, the correlation between CD24 and Siglec-10 impedes macrophage phagocytosis, thereby preventing the elimination of tumors through phagocytosis. This phenomenon contributes to an elevation in the immunological evasion of malignancies.

### 4.5 Calreticulin

Calreticulin, or CRT, functions as an "eat me" signal and is an ER lectin chaperone family member. It is crucial in triggering phagocytosis and subsequent immune responses [41,99,100]. The translocation of Calreticulin to the cell surface is prompted by cellular stress and DNA damage. When anchored to the surface of cancer cells by glycoproteins and glycans, it binds to the lipoprotein receptor-related protein 1 (LRP1) on phagocytes [41]. CRT, whether present on the surface of living or apoptotic cells, can bind with CD91, a receptor found on the surface of phagocytes. This interaction acts as a *trans*-activating signal for CD91 [60]. However, when present on the surface of phagocytes, Calreticulin also facilitates the elimination of cells that engage with CD91 in a *cis*-activating manner, thereby promoting enhanced receptor engagement. The exposure of CRT on the surface of macrophage cells relies on the Toll-like receptor (TLR)-Bruton's tyrosine kinase (BTK) pathway. This pathway triggers the phosphorylation of CRT and its subsequent secretion, leading to binding with CD91, ultimately culminating in the efficient uptake of apoptotic cells [101]. Furthermore, the phosphorylation of CRT by BTK in macrophages is a pivotal factor in the phagocytosis and removal of viable cancer cells. In essence, augmenting CRT expression on macrophages heightens the efficiency of phagocytosis. Additionally, research utilizing anti-CRT antibodies suggests that phagocytosis is driven by CRT located on the cell surface of macrophages instead of CRT on the cell surface of cancer cells. This mechanism could play a significant role in the phagocytosis of cells that lack CRT, such as red blood cells [102].

### 4.6 SLAMF and macrophage antigen 1 (Mac-1)

The Signaling Lymphocytic Activation Molecule Family (SLAMF) comprises nine single-pass transmembrane proteins, including receptors such as CD48, Ly9, CD84, and Signaling Lymphocytic Activation Molecule Family Member 7 (SLAMF7). These receptors possess 2 to 4 extracellular Ig domains and intracellular tails rich in tyrosine [103]. SLAMF7 and Mac-1 antigen interaction is vital in phagocytosis [104,105]. This interaction occurs on the surface of macrophages and triggers a signaling cascade facilitated by Src, Syk, and Btk kinases, leading to the activation of the phagocytic machinery within the macrophages [106]. Although the precise mechanism of action of SLAMF7 remains unclear, it effectively promotes the cytoskeletal reorganization necessary for phagocytosis through its interaction with MAC1 present in phagocytes [106].

### 4.7 Fc receptors

Fc receptors, which belong to a family of receptors located on the cell surface, bind to the Fc domain of immunoglobulins and initiate downstream signaling in immune cells [107,108]. Among these receptors, IgG receptors (FcγR) play a crucial role in Antibody-Dependent Cellular Phagocytosis (ADCP) of tumor cells, with FcγRIIB having an inhibitory role while others (FcγRI, FcγRIIA, FcγRC, FcγRIIIA, FcγRIIIB) function as phagocytosis-activating receptors [[108], [109], [110]]. Tumor-specific IgG antibodies opsonize cancer cells by binding to tumor antigens. Macrophages, in turn, recognize the constant region of these antibodies through FcγR expression and initiate antibody-dependent cell phagocytosis. In humans, the intracellular portion of FcγRI, FcγRIIa, FcγRIIIa, and FcγRIIIb possesses an immunoreceptor tyrosine-based activation motif (ITAM) that leads to pro-phagocytic activity. In mice, FcγRI, FcγRIII, and FcγRIV possess an ITAM [110].

## 5 Apoptotic and non-apoptotic role of phosphatidylserine; a legend of mystery

Lipids are essential components within cellular membranes [111], pivotal in upholding organismal health. Of the utmost significance are lipids as the foundational constituents of cellular membranes, with phospholipids, cholesterol, and glycolipids representing the three fundamental classes of membrane lipid molecules. Distinctive lipid compositions across the inner and outer monolayers confer disparate functions upon the two facets of a cell membrane [112].

The asymmetric distribution of diverse phospholipids within the plasma membrane of mammalian cells, initially observed in erythrocytes [[113], [114], [115]], shapes the bilayer membrane of eukaryotic cells. Among these phospholipids, Phosphatidylserine (PS), with its negatively charged head group, holds a prominent position in eukaryotic cell membranes [116]. The emergence of PS species was initially detected in lipid extracts of the brain by Folch and colleagues during the 1940s [117]. Like other phospholipids, PS features dual acyl chains at the sn-1 and sn-2 positions of the glycerol moiety, linked with the polar head group at the sn-3 position [118]. While constituting approximately 4% of all phospholipid molecules in the cell membrane, PS accounts for around 20–30% within the inner leaflet and a mere 0.2% on the exterior [119]. Perturbation of this asymmetry, leading to PS exposure on the cell surface, holds a pivotal role in apoptosis and blood clotting [116]. Consequently, the membrane's PS state emerges as a pivotal factor impacting cell survival, growth, proliferation, and manifestations associated with cancer [120].

The outward exposure of PS on the plasma membrane's exterior was first documented in 1992 [121] and recognized as an exclusive trait of apoptotic cells. Facilitating the identification and engulfment of dying cells (efferocytosis), this feature, alongside other "eat me" signals, accounts for apoptosis's immunologically silent nature. Annexin V, an anticoagulant, was previously known to bind with negatively charged phospholipids like PS, rendering it a tool for detecting PS-expressing apoptotic cells [122].

Critical to activating multiple enzymes and structural components, PS plays a pivotal role in significant events, encompassing the Clearance of apoptotic cells and the internalization of viruses into host cells [118]. Two fundamental enzymatic activities regulate the distribution of PS between leaflets. The first, an ATP-dependent enzyme known as flippase and floppase, orchestrates the bidirectional transport of phospholipids and other molecules between the outer and inner membrane leaflets. The second enzymatic activity governs the PS distribution between leaflets and catalyzes swift and non-specific phospholipid exchange across the bilayer's two facets, termed the scramblase [123].

Flippases facilitate the transverse diffusion of phospholipid molecules between the bilayer's layers, a phenomenon called "flip-flop" transition. ATP hydrolysis, driven by a P4 ATPase, propels the translocation of PS from the exoplasmic to the cytoplasmic layer. Flippase activity incites biophysical and biochemical modifications in membranes that influence cell physiology [124]. In contrast, scramblases, which are ATP-independent and non-specific, mediate bidirectional transference between the inner and outer layers [125].

Cell death, characterized as programmed (apoptosis) or accidental (necrosis, necroptosis, or pyroptosis), is instigated by factors such as tumor necrosis factor (TNF) or bacterial infection. In apoptosis, necroptosis, or pyroptosis, cells present PS on their surface, leading to phagocyte engulfment. Within the apoptotic caspase cascade, downstream caspases 3 and 7 cleave and inactivate ATP11A and ATP11C flippases. Simultaneously, caspases 3/7 cleave and activate scramblase Xkr8 [126], resulting in irrevocable cleavage and PS exposure. Cells that express PS via constitutively active scramblase retain functional flippase, leading to diminished PS receptor affinity on macrophages due to PS flipping [127]. Without flippase activity, PS remains exposed, failing to return to the inner leaflet and heightening the cell's PS receptor affinity. Due to the low inherent transition through the bilayer, disruption of phospholipid asymmetry requires time. As such, scramblases expedite rapid PS exposure during apoptosis and contribute to PS exposure in necrotic cell death. TNF-induced necroptosis and bacteria-induced pyroptosis are facilitated by calcium influx [126].

While there is definitive evidence that BAI1 can mediate PS recognition and initiate downstream signaling, recent scrutiny has cast doubt on BAI1's overall significance in macrophage biology due to its low expression levels within macrophages [128]. Various downstream signaling pathways are inevitably implicated due to many PS recognition receptors. Notably, integrins engaged in PS phagocytosis might independently activate Rho GTPases through pathways divergent from Elmo1-Dock1. This process recruits conventional integrin signaling molecules like focal adhesion kinase (FAK) and integrin-linked kinase (ILK), triggering numerous RhoGEFs downstream [129]. For instance, in endothelial-like trabecular meshwork cells, integrin αvβ5 binding activates the GEF Tiam1, critical for phagocytosis, with Dock1 playing a non-essential role [130].

Similarly, Vav3, a GEF, functions as a downstream Rho GTPase activator in macrophages, particularly for the integrin αvβ5 and direct PS receptor TIM4. Furthermore, VAV family GEFs, particularly Vav1, respond to PS involvement via TAM receptors (Tyro3, Axl, Mer), members of the receptor tyrosine kinase family. These receptors' intracellular catalytic domains directly partake in receptor kinase activity, with Mer directly binding and subsequently releasing phosphorylated Vav1 [131].

## 6 Conclusion and future perspective

Developing new strategies to control cancer progression and overcome treatment failure will likely become imperative in the coming years. It is increasingly evident that a complex interplay of inhibitory and stimulatory signals regulates tumor cells' phagocytosis and subsequent immune recognition. These signals must all be considered to elicit the most effective anti-tumor responses. While the CD47-SIRP axis was the initial phagocytosis checkpoint identified in cancer, subsequent research has revealed additional phagocytosis checkpoints. Genetic screening strategies, previously employed to identify critical regulators of phagocytosis in physiological and non-neoplastic pathological contexts, could potentially be extended to oncology. This expansion could lead to the discovery novel methods for identifying crucial phagocytosis regulators. Such an approach holds promise for uncovering new checkpoints, stimulatory molecules, and their collective impacts on cancer immune evasion. Notable benefits include enhanced efficacy, minimized side effects, and the potential for precise tumor targeting. However, associated drawbacks encompass cost, limited efficacy, and the potential for adverse immune reactions. Thus, this strategy could significantly inform the development of effective immunotherapies for cancer treatment.

Immunotherapeutic strategies involving "eat me"/"Don't eat me" signals present a promising breakthrough for various cancer treatments. Combining therapies that target these signals in cancer, alongside other targeted interventions such as Chimeric Antigen Receptor (CAR) natural killer (NK) cells and CAR-T cells, holds the potential to emerge as a novel and impactful approach for treating cancer in the future.

In conclusion, several key considerations should be highlighted. Firstly, this review solely examined the promising strategy of "eat me"/"Don't eat me" signals for cancer treatment/control, and it can be aligned with other combinational approaches to explore the collective effects of signal modulation on cancer therapy. Secondly, it is imperative to conduct a pan-cancer meta-analysis assessing the impact of representative molecules associated with "eat me"/"Don't eat me" signals on cancer patients, particularly regarding prognosis. This analysis could be facilitated by utilizing publicly available data such as The Cancer Genome Atlas (TCGA), expediting the translation of this research field into clinical applications. This endeavor is expected to engage significant readers and emphasize the significance of advancing this field into the clinic. Furthermore, from a clinical viewpoint, providing readers with insights into the current status of drug development linked to "eat me" and "Don't eat me" signals and the progress of related clinical trials holds substantial importance.

## Author Contributions

The initial draft was composed by Am.K., F.B.Y, F.F., A.K., and S.S. Figure creation was undertaken by Am.K., F.B.Y., F.V., and Am.K. F.B.Y., and M.S.G. were responsible for the research's design and study conception. M.S.G. reviewed and refined the final draft. The completion of the work garnered unanimous approval from all authors.

## Statement and declarations

### Funding

"This research did not receive any specific grant from funding agencies in the public, commercial, or not-for-profit sectors."

## Author contribution statement

All authors listed have significantly contributed to the development and the writing of this article.

## Ethical approval

Not Applicable.

## Declaration of competing interest

The authors declare that they have no known competing financial interests or personal relationships that could have appeared to influence the work reported in this paper.
